# Women’s perspectives on health facility and system levels factors influencing mode of delivery in Tehran: a qualitative study

**DOI:** 10.1186/s12978-019-0680-2

**Published:** 2019-02-08

**Authors:** Mahboube Shirzad, Elham Shakibazadeh, Ana Pilar Betran, Meghan A. Bohren, Mehrandokht Abedini

**Affiliations:** 1 Department of Health Education and Promotion, School of Public Health, Tehran University of Medical Sciences, Tehran, Iran; 2 Department of Reproductive Health and Research, World Health Organization (WHO), Geneva, Switzerland; 3 School of Population and Global Health, Centre for Health Equity, Gender and Women’s Health Unit, University of Melbourne, Melbourne, Australia; 4 Maternal Health Department, Ministry of Health, Tehran, Iran

**Keywords:** Cesarean section, Vaginal delivery, Cesarean delivery, Vaginal birth after cesarean, Qualitative study, women’s views

## Abstract

**Background:**

Iran has one of the highest national caesarean section rates worldwide. Few studies explored in-depth the health-facility and health-system level factors that affect women’s choices on mode of delivery in Iran. The aim of this study was to explore the health-facility and health-system level factors affecting women’s preferences on mode of delivery in Tehran.

**Methods:**

We conducted a qualitative study using in-depth face-to-face interviews with women between October 2017 and May 2018. Study participants were sampled purposively from a range of health service settings to include women of varying experiences regarding childbirth. Eligibility criteria were Persian-speaking, women with or without childbirth experiences. All interviews were audio-recorded and lasted 30–45 min. After verbatim transcription of the interviews, we created a preliminary thematic framework to analyze the data. A combined inductive (themes emerging from the data) and deductive (key concepts across existing frameworks) approach was used during data analysis.

**Results:**

In total, 26 in-depth interviews were conducted. Five central themes influencing women’s preferences on mode of delivery emerged from the analysis: (1) *health system conditions* (important differences between the quality of care provided at private and public hospitals; staff shortages, skills, competency, motivation and also accessibility to staff during the longer time required for a vaginal delivery; policies and protocols on vaginal birth after cesarean, pain relief for vaginal birth, and having companion during labor; (2) *standards of care in facilities* (physical condition in facilities; physical examinations and procedures; continuous and organized care; ethics); (3) *interaction between women and providers* (communicating in a supportive manner with women and communication women’s partners/families); (4) *preserving women’s dignity* (delivering high quality and respectful care); (5) *provision of information* (education about pregnancy and childbirth including environment in facility, labor processes/procedures, and the risks and benefits of both vaginal delivery and caesarean section).

**Conclusions:**

Our study suggests, there are barriers to increasing demand for and satisfaction with vaginal birth, such as women’s perceived sub-optimal quality of care during labor and birth, understaffed facilities that lack standard protocols and have limited physical space, and lack of privacy and dignity. The multifactorial nature of the increase of unnecessary Cesarean section calls for multicomponent interventions to revert this trend. These interventions need to address the health-systems’ and health-facilities’ deficiencies behind women’s preference for Cesarean section.

**Electronic supplementary material:**

The online version of this article (10.1186/s12978-019-0680-2) contains supplementary material, which is available to authorized users.

## Plain English summary

Iran has one of the highest caesarean section rates worldwide. The aim of this study was to explore the health-facility and health-system level factors affecting women’s preferences on mode of delivery in Tehran. We interviewed 26 women of varying experiences regarding childbirth between October 2017 and May 2018. Our findings showed that the following factors influence women’s preferences on mode of delivery: (1) health system conditions (important differences between the quality of care provided at private and public hospitals; staff shortages, skills, competency, motivation and also accessibility to staff during the longer time required for a vaginal delivery; policies and protocols on vaginal birth after cesarean, pain relief for vaginal birth, and having companion during labor; (2) standards of care in facilities (physical condition in facilities; physical examinations and procedures; continuous and organized care; ethics); (3) interaction between women and providers (communicating in a supportive manner with women and communication women’s partners/families); (4) preserving women’s dignity (delivering high quality and respectful care); (5) provision of information (education about pregnancy and childbirth including environment in facility, labor processes/procedures, and the risks and benefits of both vaginal delivery and caesarean section). Our study suggests, there are barriers to increasing demand for and satisfaction with vaginal birth, such as women’s perceived sub-optimal quality of care during labor and birth, understaffed facilities that lack standard protocols and have limited physical space, and lack of privacy and dignity. Lack of trust and unsatisfactory communication between women and healthcare providers are also important barriers.

## Background

When medically justified, caesarean sections (CS) have an important role in the prevention of maternal and perinatal mortality and morbidity; however, there is no evidence showing the benefits of CS for women or infants who do not require the procedure [1]. In many settings, evidence suggests a risk of poorer outcomes (such as maternal, perinatal and neonatal morbidity, and psychological or social well-being) associated with unnecessary CS [2, 3]. Furthermore, some potential complications of delivery by CS are not immediately seen after delivery, and studies have shown that the rate of hospitalization within 30 days after birth is twice more likely with a CS than with a vaginal delivery [4].

Although the World Health Organization (WHO) does not promote any specific CS rate as optimal or as a target to achieve, CS rates at population level higher than 10% are not associated with reductions in maternal or infant mortality [1, 5]. In the past few decades, however, CS has become increasingly common worldwide in both developed and developing countries [1, 5]. Between 1990 and 2014, the global CS rate increased to 12.4%; with the highest absolute increase in Latin America and the Caribbean (19.4%); the lowest in Africa (4.5%). Worldwide 18.6% of all births occur by CS, ranging from 6 to 27.2% in the least and most developed regions, respectively [5]. Iran has the highest CS rate (47.9%) in Asia [5]; almost one in two women in Iran now give birth by CS. The CS rate have increased from 35% in 2000 to 47.9% in 2016, in Iran [1, 6]. This growing number can result in many problems; from increases in healthcare costs to increases in the risk of mortality and morbidity in both mothers and neonates, which in turn affects health resources [7, 8].

Globally, it has been estimated that about 50% of CS are unnecessary [9–11]. The reasons behind the increase of unnecessary CS are multiple. These reasons are related to women or families (e.g. fear of pain, misinformation), healthcare providers (e.g. convenience, fear of litigation), health-facilities (e.g. sub-optimal supportive care), and socio-economic level (e.g. cultural trends, financial incentives). The multiple stakeholders (e.g. women, healthcare providers, facilities and organizations) involved in the decision-making process render it a complex pathway for action.

In Iran, the Ministry of Health and Medical Education (MOHME) is responsible for planning, monitoring, and supervision of health-related activities for the public and private sectors [12]. In the health system, private and public sectors both provide health care and medical services; however, public sector cover a considerable amount of population around the country [13]. According to official data, more than 90% of Iranians are under the coverage of at least one kind of health insurance [14]. Private hospitals and supplemental insurance plans support elective CS by providing high quality facilities for women. In all public hospitals, vaginal births are free of charge [14].

There has been an overall improvement in maternal and reproductive health in Iran since 1990. The maternal mortality ratio was 25 (uncertainty interval (UI) 21–31) in 2015, representing a 75% reduction since 1990 [15]. Furthermore, almost all women in Iran have at least four antenatal care visits (94%) and give birth in a health facility (95%) [16, 17]. However, there are challenges facing the country in relation to maternal health improvement including implementing standard clinical protocols for providing pregnancy, delivery and post-delivery services and promoting the quality of reproductive health services [18].

In order to ensure that childbirth services reflect women’s values and preferences, there is a need to incorporate a focus on women when designing and testing interventions aiming at reducing unnecessary CS. Women’s views and opinions should be considered so that a more comprehensive program can be designed to reduce unnecessary CSs. Several studies in Iran have reported women’s and healthcare providers’ perspectives regarding mode of delivery and their motives [19–28]. Factors that have been described in Iran include women’s fear of pain during labor and childbirth [20–22, 29]; concerns about genital modifications after vaginal delivery [23]; the belief that CS is safer for the baby [24]; and the convenience for both health professionals and the women and her family [25, 28]. Some studies have identified the reasons for high CS rates in Iran according to obstetricians and midwives [26, 27] and the impact of social and economic factors on the increase in unnecessary CS have also been documented [30]. However, these studies did not explore in depth the health-facility and health-system level factors that affect women’s choices on mode of delivery. There were no studies available to investigate women’s views on health-facility and health-systems levels that make them to choose CS. This study aimed to shed light on this part of the issue. The aim of this qualitative study was to explore the health-facility and health-system factors affecting women’s preferences for mode of delivery in Tehran. This analysis will provide an in-depth understanding of women’ views and concerns related to the quality of maternity care and other barriers embedded in the health systems. It will also advance the understanding of the underlying mechanisms at play in the decision for mode of birth in Iran. Thus the results will provide critical contextual information to inform the design of effective interventions on the targeted healthcare professionals to use the recommended behaviours in their current practice. It also can help to develop national and subnational policies and local strategies to ensure proper and high quality maternity services to women in order to reduce rates of unnecessary caesarean sections.

## Methods

This study is reported according to the consolidated criteria for reporting qualitative research (COREQ) [31] (Additional file 1: Annex 1).

### Design and participants

We conducted a qualitative study using in-depth face-to-face individual interviews with women between October 2017 and May 2018 in Tehran, Iran. Study participants were sampled purposively from a range of health service settings in Tehran to include women of varying experiences regarding childbirth being pregnant or not. These settings were the most crowded ones in the city and provide health and medical services to a large number of women. Eligibility criteria for this study were Persian-speaking (as it is the most widely spoken language in Tehran) and adult women with or without childbirth experiences (CS or vaginal delivery). We included the women with no experience of delivering a child due to the following reasons: 1) As this study is targeted at women, we needed to have a wide range of beliefs among women in different childbirth status; 2) In Iran, generally, women tend to have children; so their beliefs that can be shaped with their ‘significant others’ are important to be investigated and corrected before they arrive at childbirth stage. This could help us to design appropriate interventions on reducing unnecessary CS targeted at women with categorizing them in different experiences. We initially aimed to recruit a sample of 20 women. However, we achieved saturation of relevant themes by interviewing with 26 women.

### Data collection

Following two pilot semi-structured interviews, we developed our initial topic guide and study procedures. Women attending four maternal and/or child care clinics affiliated to the Tehran University of Medical Sciences waiting for a maternal or child healthcare service were approached to participate. Interested women were taken to a quiet private place in the clinic to proceed with the one-to-one interviews. No one else was present aside from the participant and researcher during each interview. It provided a relatively ideal place for interview. All participants were informed of the study objectives and procedures, including audio recording, and provided written consent prior to initiating the interviews. Participants were assured that they could discontinue the voice recording at any point, or refuse to answer any questions that they did not feel comfortable answering. Interviewers (M. Sh (PhD candidate) and E. Sh (Associate professor)) were female and there was no prior relationship between interviewers and participants. E. Sh has previously conducted several qualitative studies. She trained M. Sh about how to conduct interviews and supervised her. Interviews commenced with a broad introductory question, “Did your experience of childbirth (or what have you heard about it) in a health facility affect your decision on mode of future deliveries?” The interview process continued with further descriptions of the participants using prospect questions to probe for deeper perspectives using an open and interactive response. At the end of the interview, women’s demographic information was collected (i.e. age, education, self-reported socio-economic status, job status). The interviews were conducted in Persian and the duration varied between 30 and 45 min. No repeat interviews were carried out. The full interview guide is shown in Additional file 2: Annex 2. A sample key question was: can you please tell me if any system/facility condition affects your decision on mode of future deliveries; and how did your experience affect your decision?

### Data analysis

The audio recorded interviews were translated verbatim into Persian by M. Sh, and were checked for quality by E.Sh. After transcription, two authors (E. Sh and M. Sh) read the interviews and created an initial codebook of key emergent themes (investigator triangulation). Then, they tested the initial codes on the transcripts manually, agreed on a coding style and the final codebook, and coded the remaining transcripts. We also reviewed and considered existing resources to inform the organization of a preliminary thematic framework [32], which included: the WHO quality of care framework for pregnant women and newborns [33]; mistreatment of women during childbirth typology [34] and the respectful maternity care typology [35]. Thus, a combined inductive (themes emerging from the data) and deductive (key concepts across existing frameworks) approach was used during data analysis. We listed preliminary themes according to the frameworks; and then put data within these themes. We added/deleted themes based on our data from interviews and finally reached five main themes. Data were analysed in Persian, and participant quotations were translated for this paper.

In order to ensure trustworthiness of the data, the researchers used various methods, including recruiting diverse research participants. Transcription was conducted by the interviewer immediately after interviews, and the researchers discussed key learning throughout the data collection process in order to further explore emerging concepts in subsequent interviews. In order to improve the reliability of the study, the interviews were analyzed by two members of the research team independently. Due to time restrictions, the transcripts were not returned to participants for comment and/or correction.

## Results

In total, 26 in-depth interviews with women were conducted. Two women refused to participate due to their own time constraints. Table 1 presents the participant’s sociodemographic characteristics. Women were 17 to 39 years old (mean: 30.38). Sixteen women had high school education and four preferred vaginal delivery in a future hypothetical pregnancy. Five women were of high-, 17 were of middle- and four of low socio-economic status (self-reported). Only eight women were currently employed. Fifteen women had at least one previous CS.

**Table 1:** Characteristics of the participants

Sociodemographic indicator	Number (%) (*n* = 26)
Age	
< 20	2 (7.7%)
20–24	3 (11.5%)
25–29	6 (23.1%)
30–34	6 (23.1%)
35–39	9 (34.6%)
Pregnant at time of interview	
Not Pregnant	9 (34.6%)
Pregnant	17 (65.4%)
Education	
Illiterate	1 (3.8%)
High school	16 (61.5%)
University degree	9 (34.6%)
Parity	
0	7 (26.9%)
1	6 (23.1%)
2	10 (38.5%)
3	3 (11.5%)
Mode of previous delivery	
None	7 (26.9%)
Vaginal birth only	4 15.4%)
CS only	11 (42.3%)
CS & vaginal birth	4 (15.4%)
Economic class	
Low	4 (15.4%)
Middle	17 (65.4%)
High	5 (19.2%)
Currently employed	
Not employed	18 (69.2%)
employed	8 (30.8%)

Although most participants were of middle-class economic status, most stated that the cost of delivery or CS was the least importance factor in making decision by them and their families. Five central themes influencing women’s preferences on mode of delivery emerged from the interviews: health system conditions, standards of care in facilities, interaction between women and providers, preserving women’s dignity, and provision of information (Table 2).

**Table 2:** Health-facility and health-system level factors influencing women’s preferences on mode of delivery; categories, themes and subthemes

Categories/Themes	Subthemes
Theme 1: Health system conditions	
- Hospital type (public, private)	- Doing procedures by doctors or students - Stressful environment
- Staff	- Staff number - Access to staff - Competent and motivated staff
- Birth policies	- Availability of VBAC - Pain relief options in NVD - Partner/family’s companion during labor/ delivery
Theme 2: Standards of care in facilities	
- Physical condition of birth facility	- Comfortable, and calming birth environment - Clean environment during both labor and birth
- Physical examination and procedures	- Asking permission to carry out labor and childbirth procedures - painful vaginal examination - unnecessary vaginal examination
- Continuous and organized care	- Lack of neglect and abandonment - Timely care
- Ethics	- Women trust healthcare providers in recommending mode of delivery - Privacy
Theme 3: Interaction between women and providers	
- Communicating in a respectful and supportive manner with women	- Confident and supportive relationship care
- Communicating with women’s partners/families	- Providing information for women’s partners/families
Theme 4: Preserving women’s dignity	Not blaming women for their fears and/or moaning and crying due to pain - Providing women-centered care
Theme 5: Provision of information	- Information about the environment, labor processes/procedures - Information about risks and benefits of vaginal delivery and/or CS

Exemplar narratives are presented within each theme and its subthemes as follow:

### Theme 1: Health system conditions

Health systems aim to provide high quality services for maternal and child healthcare. However, the quality of care can be affected by the type of healthcare centers in terms of being private or public. Furthermore, staff shortages, skills, competency and motivation, and accessibility to staff are other factors influencing quality of care; as well as policies and protocols of healthcare systems.

#### Hospital type (public, private)

Women reported important differences between the quality of care provided at private and public hospitals. Women believed that to receive high quality care, they needed to go to private hospitals to give birth. Only in private hospitals, they were able to choose CS as a mode of delivery (maternal request). Even women who were willing to give birth vaginally specified that resource constraints in public facilities such as physical conditions would ultimately influence them go to private hospitals.

Some women complained of medical procedures conducted by medical and/or midwifery students in public hospitals during vaginal deliveries, such as vaginal examinations. They believed that the students lacked sufficient knowledge and experience to provide proper care to women. Women also mentioned that public hospitals had crowded and stressful environments, where students may make mistakes.

*“Once I was pregnant, I was referred to the emergency department in a public hospital. A medical student asked me some questions to fill my medical records. She was very tired and made many mistakes. The paper was full of crossed outs. It took a lot of time. You can’t believe … I stayed about 3 to 4 hours for my forms to be filled. One thinks it is easy to go to the operating room and undergo CS.” (Pregnant woman with CS experience; 35 years old).*

Another woman stated that:

*“Public hospitals like … I was not satisfied at all. It was very crowded and under-staffed.” (Pregnant woman without birth experience; 19 years old).*

Public hospitals do not allow CS for maternal request:

*“Public hospitals don’t allow for Cesarean birth* [on maternal request]*; but private ones do. I think it must not be banned in public hospitals” (Woman with NVD experience; 37 years old).*

#### Staff

Staff shortages and accessibility to staff during the longer time required for a vaginal delivery were mentioned as influencing factors in choosing CS as the mode of delivery. Staff were often responsible for many women in labor, and women perceived this influence their poor tempers.

*“I had a normal delivery. The baby’s head was out. Other women cried to call nurses. We were 10 patients with one nurse. How could she meet all needs or listen to us? It was inevitable that she became angry or bad-tempered. Every woman should have a nurse beside her. So cesarean is better. You don’t feel pain and such things.” (Pregnant woman with NVD experience; 26 years old).*

Staff’s skills, competency and motivation were also important factors in women’s decision for mode of delivery. They perceived doctors to be less skillful in conducting vaginal deliveries because of the increasing rates of CS:

*“For me it is very important to have a competent doctor. It will be risky if the fetus is not rotated. There will be no oxygen for the fetus. Here the role of skilled birth attendant is important. Nowadays they are not that much skillful in vaginal delivery because the cesarean is very common and they do not have practice in vaginal delivery anymore.” (Pregnant woman with NVD experience; 38 years old).*

#### Birth policies

Policies and protocols on vaginal birth after cesarean (VBAC) are almost non-existent in birth facilities in Iran. Women with a previous CS believed that they had to inevitably undergo CS again, and were unaware that VBAC was an option.

*“I had a previous Cesarean; so I could not undergo vaginal birth. I did like to have vaginal delivery. My mom had vaginal delivery too. I could do it, but it was not possible at all” (Woman with CS experience; 19 years old).*

In many of the labor wards in Iran, there are no pain relief options for women during labor. Most of the women suffer from labor pain during vaginal birth, and may view CS as a way to bypass the pain of labor.

*“Cesarean is much better. Everything is scheduled. Pain is awful in vaginal delivery” (Woman with NVD experience; 37 years old).*

Women valued to have companion during labor. They thought that if the husbands were allowed in the labor ward to provide company and support during labor and birth, many more women would decide for a vaginal birth.

*“I think the presence of the father is important during delivery. It is very supportive and relaxing. If they allow fathers come to delivery room, I think the rate of vaginal delivery will rise.” (Woman with CS experience; 36 years old).*

### Theme 2: Standards of care in facilities

Standards of care are defined as what is required in order to achieve high-quality care around the time of childbirth. Our study showed that physical condition, examination and procedures, continuous and organized care, and ethics are important factors influencing women’s decision on mode of delivery.

#### Physical condition of birth facility

Most of the women believed that lack of appropriate physical condition in facilities discouraged them from having vaginal delivery. Long stays in the labor ward under these conditions were mentioned by the women as to be avoided.

*“I was in labor ward for 3 days with other birthing women. All were stressed out. I couldn’t eat anything. There was no comfort; I witnessed their severe pain.” (Pregnant woman with both NVD and CS experience, 39 years old).*

#### Physical examination and procedures

Physical examinations and procedures were an important source of discontent and anxiety for women in labor wards. Staff did not always ask permission to carry out labor and childbirth procedures. Painful vaginal examination and poor previous outcomes were also cited by the women frequently in the interviews.

*“Every minute a midwife come and pushes her fingers into the vagina to feel the size of cervix. This is a bad feeling. CS has none of these procedures.” (woman with experience of both NVD and CS; 28 years old).*

Women disliked the physical examinations and procedures that were conducted as part of vaginal delivery, and preferred CS as a way to avoid intrusive examinations.

#### Continuous and organized care

Neglect and abandonment or long delays were reported by women as examples of low quality care that women experience in the facilities. Lack of organization and coordination between healthcare providers resulted in what women felt as chaotic care. Continuous and organized care was valued by the women.

“*Every 2 hours doctors were changed. They came and asked what is the matter with her? Again, another one came and asked for a lab test. Another one asked for other thing. It was the worst thing; no one accepted the responsibility to do the work.” (Pregnant woman with experience of both NVD and CS; 39 years old).*

*“There was a lot of chaos in the hospital. One said why you accepted her, another said her blood pressure is low, she should be hospitalized. There was a strange chaos in the labor.” (Pregnant woman with experience of both NVD and CS; 39 years old).*

Women had the perception that their care was not coordinated across different providers, which left them feeling that they were receiving sub-optimal care. They would have preferred to have the continuous support of the same provider throughout the duration of labor:

*“I needed to have a doctor beside me. Presence of doctor is very important. My first delivery had a good start but it went problematic. Doctor just came for a moment and went. I think doctor should always stay beside women. There is doctor shortage and they only take care of women giving birth in private hospitals. I was annoyed a lot. If there was a continuous care by a doctor, I surly decided to undergo vaginal delivery next time” (Woman with NVD experience; 38 years old).*

Having continuity of care throughout labor and birth may have provided some of these women with higher confidence in the quality of care provided, and removed a barrier to attempting vaginal birth.

#### Ethics

Most participants considered doctors and healthcare providers to be the most influential people in their decision about the mode of delivery. Despite this, they also reported that sometimes they could not trust their doctors because they felt providers had hidden agendas, such as a higher financial incentive to conduct CS. However, this was only a perceived belief. Women could not recognize whether they should trust their doctors on the decision made by them or not.

*“I don’t know how much difference is in the cost of vaginal delivery vs. cesarean section. May be doctors advertise for cesarean because they can earn more money. When I go to doctor I trust her. 70-80% of cesareans may occur because doctors say to women that cesarean is easier for you; whilst our mothers were comfortable with vaginal delivery” (Woman without delivery experience; 28 years old).*

A lack of privacy in crowded labor wards meant that women were often exposed to others. This was an important and commonly cited factor influencing women’s decision on mode of delivery, as they felt embarrassment and discomfort due the lack of privacy.

*“I think rooms should be separated in labor ward. At least women should be protected from others. I was ashamed a lot. My roommates were not in pain and were asleep. I was in pain and cried. I was ashamed. They looked at me, my legs. I had a bad feeling.” (Woman with NVD experience; 37 years old).*

### Theme 3: Interaction between women and providers

The establishment of a positive and trustful relationship between women, their families, and the healthcare providers that resulted in proper communication and meaningful discussions was important for women. Women perceived this to work well when the healthcare providers took time to explain their options and calm their anxieties.

“*My first delivery was vaginal delivery. I had a very good doctor. She was very nice and she supported me a lot. She was like my mom, gave me hope, and encouraged me to do vaginal delivery. When I had severe pain I said I cannot continue, I don’t want to undergo vaginal delivery. She said you are one of my best patients. It was not so, but she was encouraging me. It was perfect. She held my hand for three hours; she stood by me till I delivered my baby. She was perfect.” (Pregnant woman with NVD experience; 28 years old).*

Most women stated that their husbands prefer CS regardless of the financial costs. They indicated that their husbands worried about the effect of vaginal delivery on their body. Moreover, women stated that their husbands were not informed and updated about their situations and status in delivery room.

*“I feel that I can tolerate the birth pain if they give me a good consultation and explain about the adverse effects to my husband. Many of men don’t like vaginal delivery because of some adverse effects on vagina. If we receive proper consultation, we can make better decisions; as some of these hearings may not be scientific and are just rumors.” (Woman without birth experience; 27 years old).*

### Theme 4: Preserving women’s dignity

Dignity in maternity care means encompassing respect and autonomy during labor and birth. It largely depends on the care that women receive from their professional caregivers. Preserving women’s dignity was another factor that women valued which sometimes women felt could only being achieved by choosing to have a caesarean section.

*“Meeting the needs of women with cesarean section was better. Staff didn’t meet our needs at all. I wished I had cesarean. I said by myself if this is vaginal delivery, cesarean is much better” (Pregnant woman with NVD experience; 39 years old).*

Some women complained of harsh or rude language used by staff in the labor ward. They also were blamed by staff for their situation they were in. These women viewed CS as a way to avoid the harsh treatment by staff during labor.

Participants believed that vaginal delivery is a physiological and normal event. They anticipated that, in general, women would prefer to have a vaginal delivery if facilities delivering high quality and respectful care were the norm.

*“… Several vaginal exams, neglect … there were no one to ask how I was. They just wanted to speed the process by rupturing the water sac to get rid of us” (Woman with NVD experience; 35 years old).*

### Theme 5: Provision of information

From the women’s perspectives, provision of information and learning about birth is important and desirable. Education about pregnancy and childbirth should include considerations about the environment in facility, education about the labor processes/procedures, and the risks and benefits of both vaginal delivery and caesarean section.

*“Vaginal delivery is ok they just should inform women that these pains are normal and everything is ok.” (Pregnant woman with NVD delivery, 35 years old).*

*“My doctor didn’t ever tell me what I should do in pregnancy, especially for vaginal delivery. They should guide us on labor processes. But they don’t inform us. If someone asks me how satisfied I was in my first delivery. I’ll say 10 per cent” (Pregnant woman with NVD experience; 37 years old).*

Our study showed that a range of health-facility and health-system level factors are important for women and may influence their decision on mode of delivery. Health system conditions and standards of care in facilities, as well as confident and supportive relationship care, preserving dignity and providing information were the most important factors cited by the participant women.

## Discussion

The findings of our study revealed women’s perspectives related to health-facility and health-system level factors and how these factors influence their preferences and decision on mode of delivery in Iran. These factors should be accounted for when designing interventions for promoting vaginal delivery and decreasing the use of unnecessary CS.

Iran presents one of the highest CS rates worldwide [5]. The concerns of policy makers and health program planners about this trend and its consequences, however, have yet to be translated into effective strategies for a reduction of the unnecessary CS. In recent years, there have been numerous attempts to design, test and implement interventions for this purpose, such as the mother-friendly hospitals; the development of standard protocols for labor and birth; implementation of preparation classes for women, midwives, and gynecologists; and workshops for specialists and midwives [36–40]. Moreover, promotion of vaginal birth is one of the seven packages of Iran’s “health sector evolution policy”, a major national health policy initiated in 2014 to improve public health [36]. This package included several strategies: vaginal birth in all governmental hospitals being free of charge, improvement in physical infrastructure of labor wards to improve privacy, preparation of facilities and standards to promote and improve vaginal birth delivery methods such as water birth to reduce pain and facilitate the delivery process, preparation classes for mothers, and financial incentives for the service provider of natural delivery in governmental hospitals to encourage them to prevent unnecessary caesarean sections [36]. There were high expectations on the success of this program among Iranian policy-makers, but other initiatives might be helpful and needed to reduce unnecessary CS rates. For example, because of high numbers of first-time CS, the availability of VBAC with specially trained professionals has great potential to reduce the number of caesarean sections. However, it is not currently available in Iran.

A systematic review of non-clinical interventions for increasing the uptake and/or the success rates of VBAC indicated that however national guidelines influenced vaginal birth rates, a greater effect was seen when institutions developed local guidelines and gave individualized information to women [41]. It is suggested to develop such local guidelines in Iran.

A qualitative study was conducted in Iran to identify barriers of reducing CS rate, as perceived by obstetricians and midwives. The system barriers were identified at economic and political context level; organizational context level; social context level; individual professional level; and finally, innovation level [27].

Building trust and developing good communication between women and healthcare providers are vital in the process of decision-making for mode of delivery. Women in our study mentioned that some doctors recommended CS because of convenience or they felt there was a hidden agenda in their recommendation. On the other hand, Yazdizadeh et al. [27] reported the concerns of specialists who claimed that many women insist on a CS from the beginning of their pregnancy and if they were denied the caesarean and the vaginal delivery would develop complications, women would file a complaint against the physicians. Reconciling women’s and health providers’ factors in the increasing trend emerge as critical to achieve effective and sustainable reductions in CS.

In Brazil, with fewer and fewer vaginal deliveries conducted comes the loss of the skills to conduct vaginal delivery [42, 43]. This risk of de-skilling has also been reported in Iran [27]. Cesareans may be a preferred option in childbirth simply because the doctors are more confident of their surgical skills than their midwifery ones. This was also mentioned in our study, as some women believed that doctors do not practice vaginal delivery, and thus the patient questions the safety of this type of birth in the hands of unexperienced provider. This was also mentioned by the majority of healthcare providers in Yazdizadeh’s et al. study [27]. The participants believed that the capacity of the obstetricians and residents in conducting a vaginal delivery has reduced in recent years which coupled with the increased number of universities and teaching hospitals as well as the increased number of obstetrics residents has led to poor and insufficient education and training and thus sub-optimal quality of care.

Our study showed that perceived staff shortages during labor and birth could influence women’s preferences on mode of delivery. Staff shortages have also been reported as a barrier in health facilities in other studies [27, 44]. A study conducted by Begum et al. (2018) in Bangladesh showed that inadequate staffing at health facilities affected influenced the preferences of women [44].

Our study suggested that perceived sub-optimal quality of care during labor and birth and lack of trust were major barrier to increase demand for vaginal delivery experience. Other studies have also reported women’s perceived lack of kind, comforting, and respectful behavior towards them during labor [44, 45]; and lack of trust in maternity ward staff [46].

Despite the cultural context for vaginal birth, women mostly preferred CS due to facility-level and system-level limitations as well as negative attitudes towards vaginal birth. Our study showed that mistreatment in health facilities, was a main reason for choosing CS by women. Other studies have also reported poor quality of childbirth care in maternity health facilities [44, 47].

Interventions targeted at women to reduce unnecessary CSs need all three levels of healthcare (individual, health facility, and health system levels). Regarding health-facility and health-system level factors, a variety of changes should be considered. For example, our study showed that women valued having a labor and birth companion which is already recommended by the WHO [48]. Moreover, at the health-facility level, the provision of effective, respectful and continuous maternity care by skilled birth attendants should be assured which requires the development and integration of standards and benchmarks relating to mode of delivery.

### Strengths and limitations

To our knowledge, this is the first attempt to focus on women’s perspectives on health-system and health-facility level factors influencing their preferences on mode of delivery in Iran. Regarding the usefulness of heterogeneous sampling, which allows the researchers to obtain different perspectives from the participants, we used purposive sampling to include women of varying experiences regarding childbirth. Since this is a qualitative study, these findings may not necessarily be transferable to other settings in Iran. However, this study was conducted in Tehran where 10% of Iran’s population lives and is therefore a diverse context. Several qualitative studies have been conducted in other cities of Iran exploring women views on mode of delivery [20, 23, 49–51]. The authors of those studies mostly have focused on individual-level factors such as fear of vaginal birth and pain; and there is little knowledge on facility- and system-level factors influencing on women’s decision in these cities. However, we expect that the results found here also apply to the rest of country. Future studies can shed more light on the issue.

The results of our qualitative study align with the WHO quality of care framework for pregnant women and newborns [33]; mistreatment typology [34] and the respectful maternity care typology [35]; and highlighted the importance of these domains and frameworks in providing maternity care. We envision that our results can inform further work on developing evidence-based interventions to reduce CS rate as well as new quantitative studies on factors affecting women’s and health providers’ preferences on mode of delivery.

We understand that women may be inclined to respond in certain ways because they had come for a certain service. However, we ensured them that their responses would not affect the service they would receive and ensured confidentiality.’

## Conclusions

Our study suggests that in Iran, perceived sub-optimal quality of care during labor and birth, understaffed facilities lacking standard protocols and physical space leading women to feel deprived of privacy and dignity are barriers to increase demand for and satisfaction in the vaginal delivery experience. Lack of trust and unsatisfactory interaction between women and healthcare providers plays also an important role. The multifactorial nature of the increase of unnecessary CS and the more active role seeking CS alleged to women call for multicomponent interventions to revert this trend. These interventions need to address the health systems’ and health facilities’ deficiencies behind women’s preference for CS.

## Additional files

Additional file 1: **Annex 1**. COREQ (COnsolidated criteria for REporting Qualitative research) Checklist. (PDF 423 kb)

Additional file 2: **Annex 2**. Interview Guide. (DOCX 15 kb)

## Abbreviations

CS: Cesarean Section
VBAC: Vaginal Birth after Cesarean
WHO: World Health Organization
