# Supplementary material for: Women’s perspectives on health facility and system levels factors influencing mode of delivery in Tehran: a qualitative study
Source: Reprod Health. 2019 Feb 8;16:15. doi: 10.1186/s12978-019-0680-2 (PMC6368775; doi:10.1186/s12978-019-0680-2)
Supplement: Supplementary file 2 — Annex 2. Interview Guide. (DOCX 15 kb) [file 12978_2019_680_MOESM2_ESM.docx]

**ANNEX 2- Interview Guide**

**RESEARCH QUESTION:** What health-facility and health-system level factors affect women’s preferences on mode of delivery?

**INTERVIEW QUESTIONS:**

- **Neutral initial question:**

Did your experience on childbirth (or what have you heard about it) in a birth facility affect your decision on mode of future deliveries

- Can you please tell me if any system/facility condition affect your decision on mode of future deliveries?
  - Can you give me an example of what you mean?
  - *Probe conditions such as hospital, staff, birth approaches, physical conditions, communication, and information.*
- How did your experience affect you decision?
- Have we missed something you think is important in making your decision?
- At the end, is there anything you would like to add?
